# Establishment of a yeast-based VLP platform for antigen presentation

**DOI:** 10.1186/s12934-018-0868-0

**Published:** 2018-02-05

**Authors:** David Wetzel, Theresa Rolf, Manfred Suckow, Andreas Kranz, Andreas Barbian, Jo-Anne Chan, Joachim Leitsch, Michael Weniger, Volker Jenzelewski, Betty Kouskousis, Catherine Palmer, James G. Beeson, Gerhard Schembecker, Juliane Merz, Michael Piontek

**Affiliations:** 1 ARTES Biotechnology GmbH, Elisabeth-Selbert-Straße 9, 40764 Langenfeld, Germany; 2 Laboratory of Plant and Process Design, Technical University of Dortmund, Emil-Figge-Straße 70, 44227 Dortmund, Germany; 3 Institute for Anatomy I, Düsseldorf University Hospital, Moorenstraße 5, 40225 Düsseldorf, Germany; 4 Burnet Institute for Medical Research and Public Health, 85 Commercial Road, Melbourne, VIC 3004 Australia

**Keywords:** Virus-like particles, Chimeric virus-like particles, Antigen presentation, DHBV, Animal infectious diseases, *Hansenula polymorpha*, *Pichia angusta*, Antibiotic-free

## Abstract

**Background:**

Chimeric virus-like particles (VLP) allow the display of foreign antigens on their surface and have proved valuable in the development of safe subunit vaccines or drug delivery. However, finding an inexpensive production system and a VLP scaffold that allows stable incorporation of diverse, large foreign antigens are major challenges in this field.

**Results:**

In this study, a versatile and cost-effective platform for chimeric VLP development was established. The membrane integral small surface protein (dS) of the duck hepatitis B virus was chosen as VLP scaffold and the industrially applied and safe yeast *Hansenula polymorpha* (syn. *Pichia angusta*, *Ogataea polymorpha*) as the heterologous expression host. Eight different, large molecular weight antigens of up to 412 amino acids derived from four animal-infecting viruses were genetically fused to the dS and recombinant production strains were isolated. In all cases, the fusion protein was well expressed and upon co-production with dS, chimeric VLP containing both proteins could be generated. Purification was accomplished by a downstream process adapted from the production of a recombinant hepatitis B VLP vaccine. Chimeric VLP were up to 95% pure on protein level and contained up to 33% fusion protein. Immunological data supported surface exposure of the foreign antigens on the native VLP. Approximately 40 mg of chimeric VLP per 100 g dry cell weight could be isolated. This is highly comparable to values reported for the optimized production of human hepatitis B VLP. Purified chimeric VLP were shown to be essentially stable for 6 months at 4 °C.

**Conclusions:**

The dS-based VLP scaffold tolerates the incorporation of a variety of large molecular weight foreign protein sequences. It is applicable for the display of highly immunogenic antigens originating from a variety of pathogens. The yeast-based production system allows cost-effective production that is not limited to small-scale fundamental research. Thus, the dS-based VLP platform is highly efficient for antigen presentation and should be considered in the development of future vaccines.

## Background

Since the 1980s virus-like particles (VLP) have been known for their immunogenic properties [1] and have been well established as safe, effective vaccines and drug delivery systems in humans [2–4]. VLP induce strong humoral immune and T cell responses but they lack the risks of conventional vaccines: they do not contain genetic material and are unable to replicate [5–7]. Recombinant VLP are highly valued as vaccine development platforms and VLP scaffolds are used to display immunogenic antigens originating from foreign pathogens (referred to as chimeric VLP) [8].

Vaccination plays a leading role in preventing infectious diseases in animals and improving animal welfare [9]. Aside from economic advantages, vaccination allows the reduced use of antibiotics in animal farming and thus helps to prevent the spread of antibiotic resistances in the environment [10]. In the veterinary sector, conventional vaccines are still predominant. Subunit vaccines based on soluble, monomeric proteins often have limitations regarding immunogenicity which can be optimized by VLP-based approaches [11, 12].

Our current study describes the establishment of a novel and versatile *Hansenula*-based VLP platform. We chose the membrane integral small surface protein (dS) of the duck hepatitis B virus (DHBV) as scaffold protein for chimeric VLP production [13, 14]. It allows the development and high-yield production of chimeric VLP which tolerate the incorporation of a variety of large foreign antigens. Thus, key challenges for VLP-based vaccine development are addressed and fulfilled using this platform, which has not been reported for other VLP platforms before [6, 15, 16].

The DHBV is closely related to the human hepatitis B virus (HBV) and the virions are of comparable size (42–50 nm in diameter) and structure [17]. However, size and composition of their subviral particles are differing which certainly induces differences in their recombinant counterparts, too. The naturally occurring VLP from the DHBV are described as 35–60 nm particles [18] and the ratio of the large to the small DHBV surface proteins within the VLP is identical (approximately 1:4, [19, 20]) to that found in the virions’ envelope [21]. In contrast, the spherical HBV VLP are smaller (~ 22 nm diameter) and the small surface protein (HBsAg) is enriched compared to the composition of the virions’ envelope [21]. Additionally, dS VLP are lacking an equivalent antigen to the highly immunogenic “a determinant” of the HBsAg that predominates the host’s immune reaction [20, 22, 23].

As a microbial cell factory, we chose the methylotrophic yeast *Hansenula polymorpha* (*H.* *polymorpha*, syn. *Pichia angusta*, *Ogataea polymorpha*, [24]). In the field of single-layer VLP production, advantages of yeast-based systems over mammalian [25, 26], bacterial and baculovirus/insect cell systems [27, 28] are widely known. In particular, *H. polymorpha* is established as safe microbial cell factory for recombinant products that have been granted “generally recognized as safe” (GRAS) status and for the production of biopharmaceuticals like hepatitis B VLP vaccines [29–31].

Another focus of this project was the development of a VLP platform suited for the production of VLP-based vaccines suitable for the application in the veterinary sector. Hence, it is compatible with the “differentiating infected from vaccinated animals” (DIVA) strategy and independent of antibiotic resistance genes during all stages of development and production [32].

Firstly, plain dS VLP (without a foreign antigen displayed) were purified at several mg scale as a proof-of-principle and benchmark. For this purpose, a downstream process (DSP) approved for hepatitis B vaccine production from yeast [31] was applied. Analytical tools for specific detection of dS, quantification of host cell protein (HCP) impurities and VLP characterization were established in parallel. Antigens of up to 412 amino acids (aa) were chosen to be incorporated in the dS VLP scaffold to test the versatility of the platform for chimeric VLP production. The chosen antigens originated from the following four different viruses that cause infectious diseases in animals:

1. The bovine viral diarrhea virus (BVDV) is an important pathogen of cattle, also infecting sheep and pigs. It is responsible for significant animal suffering and economic losses worldwide [33].
2. The classical swine fever virus (CSFV) is acknowledged as a global threat for swine [34] and is listed as notifiable animal diseases by the World Organization for Animal Health.
3. The feline leukemia virus (FeLV) is a retrovirus threatening domestic cats [35].
4. The west nile virus (WNV) is a mosquito vector transmitted zoonotic virus of the *Flaviviridae* family. It circulates in birds as natural hosts but can be transmitted to mammals including humans causing west nile fever [36]. WNV could represent a case example because of its close relationship to the yellow fever and dengue virus, which cause two of the most important mosquito-borne human diseases [37].

Antigen-presenting chimeric VLP were rationally engineered by genetic fusion of foreign antigens to either the C- or N-terminus of the dS. Co-expression of the fusion proteins with the VLP-forming scaffold protein allowed the isolation of chimeric VLP in all cases. Compared to other chimeric VLP platforms, no linker [38] or chemical coupling of the antigen to the VLP scaffold [39] was required. Thus, the use of the dS allowed us to minimize the complexity of the chimeric VLP to the essentials.

The methodology applied for purification of plain dS VLP could widely be transferred to chimeric VLP displaying the different foreign antigens. A variety of analyses regarding particle structure and stability were performed for different VLP preparations. For chimeric VLP, a shelf life of at least 6 months and resistance to temperature-induced stress comparable to that of plain dS VLP were demonstrated.

## Methods

### Genes, vectors, cloning

The designed open reading frames (ORF) encoding the dS (Genbank accession number: MF510122) and the different fusion proteins were synthesized by GeneArt/Life Technologies (Regensburg, Germany). They were flanked with *Eco*RI and *Bam*HI restriction sites and codon-optimized for recombinant expression in *H. polymorpha*. Genbank accession numbers of donor sequences are given in Table 2. Synthesized ORF were inserted between the *Eco*RI and *Bam*HI sites of the antibiotic resistance marker free *H. polymorpha* expression plasmid pB14 [40] or a derivative thereof with *LEU2* instead *URA3* gene for selection in yeast. Cloning was done in an *E. coli* K12 derivative (genotype: F-pyrF74:Tn5 supE44 lacY1 ara-14 galK2 xyl-5 mtl-l leuB6 proA2 hsdS20 recA13 rpsL20 thi-1 lambda-) purchased from DSMZ (No. DSM 6201, Braunschweig, Germany). It is optimized for cloning of yeast shuttle vectors containing *LEU2* and/or *URA3.* Chemically competent bacteria were transformed by a heat shock protocol (60 s, 40 °C [41]). For plasmid amplification, strains were grown at 37 °C in M9-based minimal medium [42] supplemented with amino acids (mg L^−1^). *l*-Arginine (10), *l*-histidine (5), *l*-isoleucine (30), *l*-leucine (30), *l*-methionine (5), *l*-proline (20), *l*-threonine (25), *l*-tryptophan (20), *d/l-*phenylalanine (30), *l*-lysine-monohydrate (20) and *l*-leucine (30).

### Heterologous yeast strain generation

The auxotrophic *H. polymorpha* strains ALU3 (relevant genotype: *ade1*, *leu2*, *ura3*) [43] and RB11 (relevant genotype: *ura3*) [43] were used as expression hosts. They are derivatives of wild type strain ATCC^®^ 34438™ (CBS 4732, IFO 1476, JCM 3621, NBRC 1476, NCYC 1457, NRRL Y-5445) [44]. Yeast transformation was performed by electroporation [45] and subsequent strain generation and isolation [46]. Thereby, the expression plasmids integrated genomically stable in different copy numbers into the host genome. Heterologous yeast strains were stored as glycerol stocks at − 80 °C.

### Expression studies and VLP diagnosis

Screening for heterologous *H. polymorpha* production strains was performed at 37 °C in 3 mL test tube scale. Pre-cultures were grown in YPD medium to stationary phase and used to inoculate YPG medium containing 20 g L^−1^ glycerol (AppliChem, Darmstadt, Germany) as carbon source. After a derepression phase of 56 h, 1% (v/v) methanol (AppliChem, Darmstadt, Germany) was added and cultivation was extended for additional 24 h. Cells were harvested by centrifugation (6000*g*, 15 min, 4 °C) and disrupted by glass beads (0.5–0.7 mm, Willy A. Bachofen, Nidderau-Heldenberg, Germany) in 1.5 mL reaction tubes on a shaker (basic Vibrax^®^ shaker, IKA^®^-Werke, Staufen, Germany) at maximal frequency for 30 min at 4 °C.

To analyze whether the fusion proteins and the dS co-expressed in *H.* *polymorpha* are involved in chimeric VLP formation, two subsequent ultracentrifugation steps were accomplished in Optima™ L90K centrifuge (rotor type: 70.1 Ti, tubes: 16 * 76 mm, Beckman Coulter, Brea, California, USA). After cell disruption, the soluble protein fractions were prepared and layered on top of a sucrose cushions (2 mL 70% (w/v); 3 mL 20% (w/v), [47]). The boundary layers between the two sucrose layers were harvested after ultracentrifugation (90 min, 51,000 rpm, 18 °C). These fractions were subsequently mixed with 6 M CsCl (AppliChem, Darmstadt, Germany) stock solution to 1.5 M final CsCl concentration. Mixtures were subjected to density gradient separation (65 h at 48,400 rpm, 4 °C). Thereafter, 11 fractions were collected according to their densities and analyzed by Western blot to specifically identify the product containing fractions. As indication for chimeric VLP formation were regarded: (1) accumulation of the product proteins in the boundary layer of the sucrose cushion ultracentrifugation. (2) Co-separation of the dS and the respective fusion protein from contaminating HCP. (3) Gravimetrically determined densities of 1.1–1.2 g cm^−3^ of the product containing fractions.

### Heterologous protein production and purification of VLP at laboratory scale

Cell mass used for pilot VLP production process was generated in a 2.5 L scale fed-batch fermentation using a stirred tank (Labfors 5, Infors, Bottmingen, Switzerland). The bioreactor was sterilized by autoclaving after filling with 2.5 L animal component free complex medium containing 20 g L^−1^ yeast extract (BD Biosciences, Heidelberg, Germany), 40 g L^−1^ peptone from soymeal (AppliChem, Darmstadt Germany), 20 g L^−1^ glycerol, 3.4 g L^−1^ yeast nitrogen base (YNB, Becton, Dickinson Difco™, Franklin Lakes, USA), 10 g L^−1^ ammonium sulfate (AppliChem, Darmstadt Germany), 0.5 g L^−1^ adenine and 2 g L^−1^ leucine. Aqueous solutions of NH_3_ (12.5% (w/w), sterile filtered) and H_3_PO_4_ (28% (w/w), Merck, Darmstadt, Germany) were used as corrective media to keep pH constant (set point 6.2) throughout fermentation. Struktol J 673 (10% (v/v) aqueous solution, Schill + Seilacher, Hamburg, Germany) was utilized as antifoam agent. Temperature was kept constant at 37 °C, aeration was adjusted to 1 vvm (2.5 NL min^−1^) and the pO_2_ setpoint was 40%. The medium was inoculated using shake flask pre-cultures. After a batch phase of 12 h, 360 mL of 750 g L^−1^ glycerol solution were fed continuously over 37 h. Target gene expression was induced by pulse-wise addition of 65 mL 28.5% (w/w) glycerol and 71.5% (w/w) methanol solution. Cells were harvested after 70.5 h total fermentation time by centrifugation (30 min, 4 °C, 17,000*g*), washed with wash buffer (50 mM Na-phosphate buffer, 2 mM EDTA, pH 8.0) and stored at − 20 °C until further processing.

The dry cell weight (dcw) was quantified using a moisture analyzer (MLS 50-3 HA250, Kern & Sohn, Balingen, Germany). OD_600_ of cell suspensions was determined with a spectrophotometer (DU 640 Beckman Coulter, Brea, California, USA).

Plain dS VLP and chimeric VLP with the dS as scaffold were purified by a DSP invented for purification of HBsAg VLP [31] including adjustments due to down-scaling of the process to laboratory scale. Briefly, PEG_6000_ and NaCl (AppliChem, Darmstadt, Germany) were added to crude cell lysate after yeast cell disruption by six cycles of high pressure homogenization (~ 1500 bar, APV 2000, SPX Flow Technology, Unna, Germany) in presence of 2 mM PMSF. The mixture was incubated over-night at 4 °C and then centrifuged (17,000*g*, 30 min, 4 °C). Subsequently, 15 g L^−1^ fumed silica matrix Aerosil (type 380 V, Evonik, Essen, Germany) was added to the soluble protein fraction (PEG-SN). Product adsorption to Aerosil was allowed over-night at 4 °C during incubation on magnetic stirrer MR3001 (Heidolph Instruments, Schwabach, Germany). The matrix was washed with 77 mM NaCl aqueous solution volume-normalized to the PEG-SN. A buffer for desorption of the product from the Aerosil was added (10 mM di-sodium tetraborate decahydrate, 2 mM EDTA, 6 mM deoxycholic acid sodium salt, pH 9.1) using a quarter of the PEG-SN volume. The suspension was stirred for 1 h at 55 °C. Only in the case of plain dS VLP, the soluble product fraction (desorbate) was applied to anion exchange chromatography (Mustang Q XT, PALL Life Sciences, Port Washington, New York, United States) and eluted with 0.5 M NaCl. Product containing fractions were pooled and concentrated by ultrafiltration (Vivaspin^®^ sample concentrator, MWCO 100 kDa, Sigma-Aldrich, Steinheim am Albuch, Germany) and applied to CsCl density gradient separation as a final purification step. Product containing fractions were pooled, desalted by dialysis (Slyde-A-Lyzer™ dialysis cassettes, MWCO 20 kDa, Thermo Fisher Scientific, Waltham, USA) against desalting buffer (8 mM Na-phosphate buffer pH 7, 154 mM NaCl, AppliChem, Darmstadt, Germany) and sterile filtered (Filtropur S 0.2 filters, Sarstedt, Nümbrecht, Germany).

For chimeric VLP preparations, the desorbate was concentrated by ultrafiltration (Minimate™ TFF tangential flow filtration Capsule Omega 100 k Membrane, PALL, Port Washington, New York, United States) and directly applied to CsCl density gradient separation.

### Particle characterization

Particle size distribution of VLP preparations was analyzed by dynamic light scattering (DLS) using a DelsaMax CORE (BCI-3161-DMC) system operating at 25 °C and equipped with a 100 mW 658 nm diode laser along with disposable cuvettes (Beckman Coulter, Brea, California, USA). Presented data are mean values from 10 acquisitions. Stability assessments at elevated temperatures were completed by step-wise increase of chamber temperature by 5 °C. Before collecting data as described before, temperature equilibration was allowed for of 5 min. Increase of temperature was continued until aggregation was detected.

Transmission electron microscopy (TEM) was used for analysis of the shape and integrity of the VLP. Volumes of 15 µL fixative (4% paraformaldehyde, 0.1 M cacodylate buffer, pH 7.2) were mixed with 15 µL of purified VLP samples. Mixtures were incubated for 15 min at room temperature (RT). Then, 3 µL of the mix were transferred to a nickel grid coated with Formvar and carbon. After 2 min of incubation at RT, the remaining liquid was removed carefully with absorbent paper and the grid was washed twice with 30 µL of distilled water and equilibrated with 30 µL staining solution (1.5% (w/v) uranyl acetate aqueous solution). The liquid was immediately removed, and the samples were stained by incubating the grids for 30 s with 30 µL staining solution. After drying at RT for at least 30 min, TEM images were generated with H600 TEM (Hitachi, Tokyo, Japan) at 75 kV.

Super-resolution microscopy (structured-illumination microscopy; N-SIM) was used to investigate co-localization and surface exposure of the scaffold protein dS and the foreign antigen in nano-scale structures. Chambered slides (Nunc) were coated with 0.01% poly-*l*-lysine (Sigma-Aldrich, Steinheim am Albuch, Germany) for 20 min before washing thrice with PBS. Native VLP samples were added to the coated wells. They were allowed to settle over-night at 4 °C. The supernatant of unbound VLP were removed and samples were fixed with 4% paraformaldehyde for 20 min before washing thrice with PBS. Samples were blocked with 6% bovine serum albumin (Sigma-Aldrich, Steinheim am Albuch, Germany) for 20 min and washed thrice with PBS. Samples were dual-labeled with primary antibodies biotinylated anti-dS mAb (7C12) and anti-CSFV E2 mAb (PrioMab CSFV V8 Monoclonal Antibody, Thermo Fisher Scientific, Waltham, USA) and subsequently, secondary labeled with streptavidin-488 (Invitrogen, Carlsbad, California, USA, green fluorescence) and anti-mouse AlexaFluor 594 (red fluorescence, Invitrogen, Carlsbad, California, USA). Samples were subjected to another fixation step with 4% paraformaldehyde for 10 min. The super-resolution images were collected using a Nikon N-SIM microscope equipped with 488, 561 and 640 nm lasers, an Andor iXON DU897 EM-CCD camera and a oil immersion lens (100-fold magnification) having a numerical aperture of 1.49. The z-series was acquired using NIS-Elements and analysed both using NIS-Elements and the open java source, ImageJ/FIJI.

### Quantification of proteins and lipids

Protein concentrations were determined by precipitation Lowry protein assay [48]. Samples were analyzed at least as triplicates. Commercial BSA stock solution (Sigma-Aldrich, Steinheim am Albuch, Germany) was used as standard. Lipid content of VLP preparations was determined based on sulfo-phospho-vanillin reaction [49] with refined soya oil (Caesar & Loretz GmbH, Hilden, Germany) used as standard.

### SDS-PAGE, Western blot and dot blot analysis

Sodium dodecyl sulfate polyacrylamide gel electrophoresis (SDS-PAGE) was used to separate proteins according to their size. The Criterion™ Cell system was used (BioRad, München, Germany). Consumables were Criterion™ XT precast gels (4–12% Bis–Tris), XT reducing agents, XT sample buffer and Precision Plus Protein™ Prestained Standard All Blue as molecular weight (MW) standard. Polyacrylamide (PAA) gels were either stained with Coomassie dye [50] or subjected to Western blot analysis for specific detection of VLP proteins. The proteins were transferred onto cellulose nitrate membranes (Sartorius Stedim Biotech, Göttingen, Germany) by semi dry blotting [51]. Prior to immunostaining, the membrane was incubated with Ponceau S solution (AppliChem, Darmstadt Germany) for reversible, non-specific staining of the transferred proteins. After de-staining in PBS and blocking of the membranes, the monoclonal antibodies listed in Table 1 were used as primary antibodies. The detection system was completed with appropriate secondary antibodies (BioRad, München, Germany) conjugated with alkaline phosphatase along with BCIP-NBT solution (VWR international, Radnor, USA). The reactivity of native VLP samples was assessed in dot blot assays without any denaturing treatment and applied to a nitrocellulose membrane by vacuum. The membranes were immune stained as described for Western blot analysis. A commercial software package was used for analysis by densitometry (Image Lab™, BioRad, München, Germany).

**Table 1:** List of monoclonal antibodies used for specific detection of the target proteins

Antigen	Primary antibody	Source
dS	7C12^a^	BioGenes GmbH, Berlin, Germany
BVDV E2	WB166	APHA Scientific, Addlestone, United Kingdom
CSFV E2	PrioMab CSFV V8	Thermo Fisher Scientific, Waltham, USA
WNV E	Ab00676-23.0	Absolute Antibody, Oxford, United Kingdom

^a^Detects the wild-type dS and the dS domain of the fusion proteins [19, 75]

### Analysis of HCP

Host cell protein (HCP) content of VLP preparations was analyzed by anti-HCP Western blot and enzyme-linked immunosorbent assay (ELISA). A polyclonal antiserum isolated from goats immunized with *H. polymorpha* HCP (Artes Biotechnology, Langenfeld, Germany/BioGenes, Berlin, Germany) was used in both cases as primary immunoreagent. The detection system for Western blot analysis was completed with a rabbit anti-goat IgG AP conjugate (BioRad, München, Germany) in combination with BCIP-NBT solution.

HCP quantification was done by an indirect ELISA in high binding plates (Sarstedt, Nümbrecht, Germany). Crude cell extract of a *H. polymorpha* vector control strain was used for calibration. The ELISA plate was first coated with the samples under investigation and then immunodecorated with the anti-HCP serum. Subsequently an enhanced streapavidin/biotin system was employed: the ELISA plates were incubated with biotinylated anti-goat polyclonal antibodies raised in rabbits (KPL, Milford, Massachusetts, USA) as secondary antibody. Then, streptavidin-HRP (GE Healthcare, Amersham, UK) was added and ABTS substrate solution was used for colorization (BioRad, München, Germany).

### Protein deglycosylation assay

*N*-Glycosylation of the heterologous target proteins was analyzed by treatment with an endoglycosidase H (EndoH) prior to SDS-PAGE and Western blotting. Protein samples were denatured (95 °C for 5 min) in glycoprotein denaturing buffer (New England Biolabs, Frankfurt a. M., Germany) and subsequently treated with EndoH in glyco3 buffer (New England Biolabs, Frankfurt a. M., Germany) at 37 °C for 60 min. A shift of target protein-specific signals in Western blot analysis to lower apparent MW compared to the untreated sample indicated *N*-glycosylation of the target protein.

## Results

### Design of fusion proteins for chimeric VLP production

Typically, co-production of the VLP scaffold protein dS and fusion proteins composed of a foreign antigen C- or N-terminally fused to the dS is required for chimeric VLP production. To demonstrate it with an application-oriented focus, a variety of fusion proteins were designed harboring immunogenic antigens originated from the animal infecting viruses BVDV, CSFV, FeLV or WNV (Table 2).

**Table 2:** Summary of fusion proteins constructed and recombinantly produced in *H. polymorpha*

Viral source protein (Genbank accession number)	aa used for fusion protein (number of predicted *N*-glycosylation sites^a^)	Fusion protein designation	C- or N-terminally fused to dS	Number of aa^b^	MW [kDa]	Signal sequence (CL) or start methionine (M) included	Genbank accession number for the fusion protein coding gene
BVDV E2 (AEV54362.1)	1–344 (4)	E2BVDV344-dS	N	511	57.5	CL	MG280712
	1–196 (2)	E2BVDV196-dS	N	363	40.3	CL	MG280713
	1–196 (2)	dS-E2BVDV196	C	363	40.3	M	MG280714
CSFV E2 (AAT85717.1)	1–337 (5)	E2CSFV337-dS	N	504	56.1	CL	MG280715
	1–184 (2)	E2CSFV184-dS	N	351	38.7	CL	MG280716
	1–102 (0)	E2CSFV102-dS	N	270	29.5	M	MF510123
FeLV env (AAA43051.1)	1–412 (9)	FeLVp45-dS	N	580	63.7	M	MG280717
	1–132^c^ (0)	FeLVp15E-dS	N	326	35.8	M	MG280718
WNV E (ADI33161.1)	293–454 (0)	EDIIIWNV-dS	N	330	35.3	M	MG280719

^a^Predicted by NetNGlyc 1.0 [76]

^b^167 aa of the dS included, mature protein

^c^Including the single aa exchange E82R [77]

The envelope protein E2 appeared as valuable antigen for targeting BVDV and CSFV. For both viruses, E2 was described to be the key immunogen involved in neutralization upon infection [52–55]. To vary the complexity of the constructed fusion proteins, N-terminal parts of the respective E2 of different lengths were chosen to be displayed on the VLPs’ surface. For the fusion protein design, information on structures and immunogenic domains was considered [56–58]. In the longer fusion protein variants which contained *N*-glycosylation motifs, the leader peptide of the chicken lysozyme (CL) was included. Thus, fusion proteins were targeted to the secretory machinery of the yeast [59] which enhanced protein *N*-glycosylation of the constructs to improve their immunogenic potential [60].

The protein p45, especially in combination with p15E, was reported to protect cats from FeLV infection [61–63]. Both antigens were included in our project as well as domain III of the WNV envelope protein E which is also known as potent immunogen [64].

### Co-expression of the dS and the designed fusion proteins

Aiming at co-expression of the dS and the fusion proteins for recombinant chimeric VLP production, the following three strain generation strategies were applied. The constructed expression plasmids were based on pB14 which is free of antibiotic resistance genes [40].

**Fig. 1:**
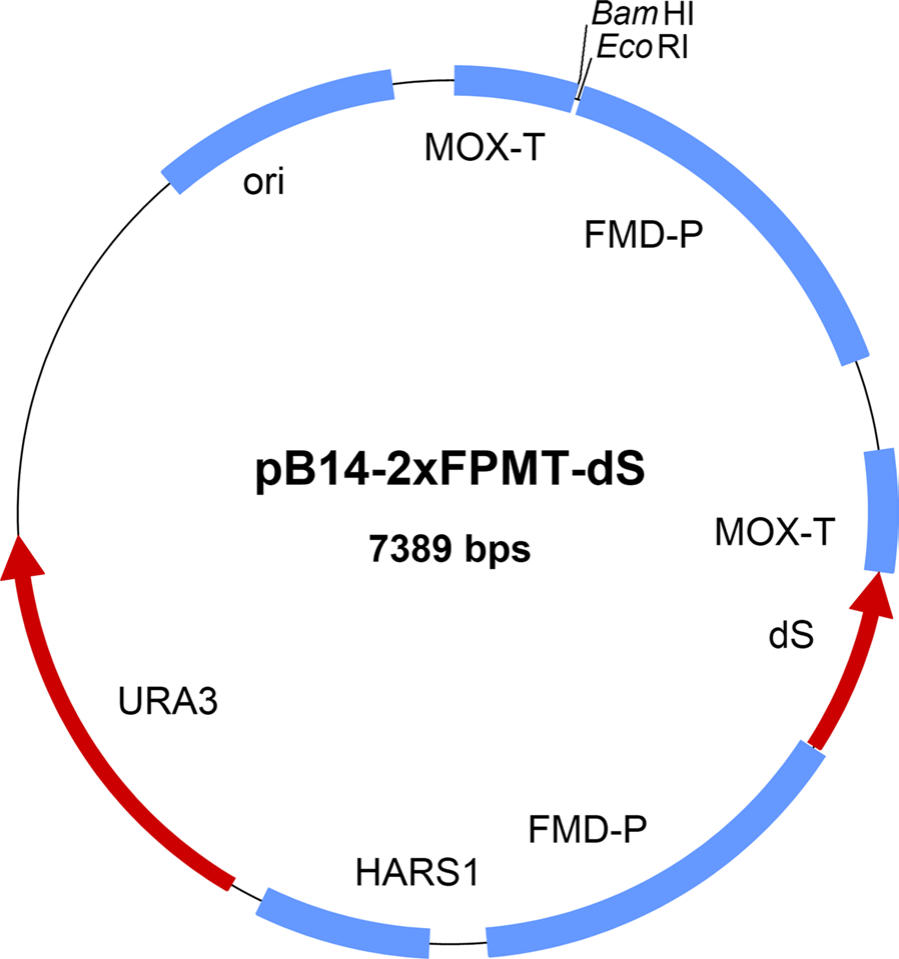
Map of the novel expression plasmid pB14-2xFPMT-dS carrying two expression cassettes specifically tailored to heterologous co-production of the dS and a fusion protein for chimeric VLP production with *H.* *polymorpha*. The dS encoding gene is stably inserted. The ORF encoding the desired fusion protein is to be inserted using *Eco*RI and *Bam*HI sites. The *S. cerevisiae* URA3 gene was used for selection of bacteria and yeast. Further features: ori, origin of replication; HARS1, *H. polymorpha* autonomously replicating sequence 1; FMD-P, *FMD* promoter; MOX, *MOX* terminator both derived from *H. polymorpha* genome

I. Staggered transformation: firstly, a dS-encoding expression plasmid was introduced into ALU3 and a strain producing dS was isolated and cryo-preserved at − 80 °C. In a second transformation, an expression plasmid encoding the fusion protein of choice was introduced into the selected dS-producing strain. Strains co-producing dS and the fusion protein could then be isolated.
II. Co-transformation: ALU3 was transformed in one electro-transformation with two pB14-based plasmids or plasmid fragments, respectively. One of them encoded the dS the other encoded the respective fusion protein. The plasmids or plasmid fragments carried unequal selection markers.
III. Dual plasmid approach: transformation of ALU3 or RB11, respectively, with the novel pB14-2xFPMT-dS expression plasmid (Fig. 1) encoding both, the dS and a fusion protein after insertion of an appropriate gene.

**Fig. 2:**
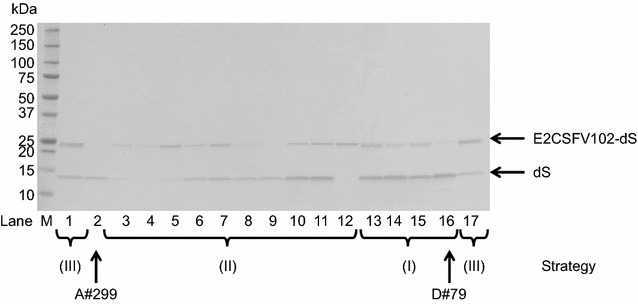
Western blot analysis of crude cell lysates probed with anti-dS mAB 7C12. Samples originated from 17 different recombinant *H. polymorpha* strains co-producing the dS and the fusion protein E2CSFV102-dS (lanes 1, 3–17) or producing only the dS (lane 2, strain A#299). Indicated strain generation strategies (I), (II) and (III) refer to the methods described in the text. Strains A#299 and D#79 (highlighted by arrows) were used for VLP production. M, molecular weight marker

All three strain generation strategies yielded strains co-producing the dS and each of the designed fusion proteins (Table 2). The dS and the dS domain of the fusion protein respectively, were specifically detected by anti-dS Western blots. This is exemplarily shown for co-production of dS and E2CSFV102-dS in Fig. 2. Strains producing both heterologous proteins at approximately equal levels (lane 7) could be identified as well as strains producing one of them in excess (e.g. lanes 1 and 16). The dS expression levels of the strains generated by transformation with pB14-based plasmids without an antibiotic resistance gene reached (e.g. lane 1) or surpassed (e.g. lane 16) the productivity of the reference strain A#299 (lane 2) generated with a pFPMT121-based plasmid encoding the dS. Finally, for each fusion protein one strain has been selected and used for further analyses. Designated strains are listed in Table 4. Transformation of strain M#22-8 with a FeLVp15E-dS encoding expression plasmid yielded strain M#4-5 which co-expresses the three heterologous proteins dS, FeLVp45-dS and FeLVp15E-dS.

Each of the strain generation strategies (I), (II) and (III) yielded heterologous yeast strains which stably co-produced the dS and a fusion protein within only one experimental sequence of transformation and selection. However, the strategies had their individual advantages summarized in Table 3. The staggered transformation approach (I) reduced lab work to a minimum since only a small state-of-the art expression plasmid encoding the fusion protein needed to be assembled and verified by sequencing. Also, transformation efficiency and frequency of strains co-producing the two desired proteins were maximal. The greatest variety of expression levels of the two target proteins was observed among strains isolated from strategy (II). However, transformation efficiency was lower compared to strategy (I) and (III): two plasmids with two unequal selection markers were used in the same transformation and only transformants having both plasmids incorporated were selected. For strategy (III) only one auxotrophy marker (*ura3*) was needed. This expanded the choice of applicable host strains to e.g. the industrially established strain RB11. Transformation of strain RB11 with the dual plasmid (Fig. 1) yielded directly prototrophic production strains. Nevertheless, the large plasmid harboring multiple homologous sequences (*FMD* promoter, *MOX* terminator, dS encoding sequence, Fig. 1) complicated cloning work and especially sequencing of the ORF encoding the fusion protein.

**Table 3:** Qualitative characterization of the three strain generation strategies described in the text

	Strategy (I): staggered transformation	Strategy (II): co-transformation	Strategy (III): dual plasmid approach
Speed of lab work	+++	++	++
Host strain options	+	+	++
Transformation efficiency	+++	+	+++
Frequency of positive strains^a^	+++	++	+
Variety of productivity among positive strains^a^	+	+++	+

^a^Positive strains: strains producing both heterologous proteins, the dS and the fusion protein

### Solubilization of the target proteins

Interestingly, in case of co-expression of dS and E2CSFV102-dS, the relative expression levels of both target proteins had an impact on their solubilization during cell disruption. If the dS was produced in excess over E2CSFV102-dS more than 80% of both heterologous proteins were found solubilized in the supernatant after cell disruption. However, if the protein levels were equal or if the fusion protein was produced in excess, both product proteins were found mainly insoluble (data not shown). In the DSP for VLP purification adopted from HBsAg VLP vaccine production [31], the supernatant after cell disruption is processed. Due to the improved product solubilization in strains producing the dS in excess over E2CSFV102-dS, strain D#79 (Fig. 2, lane 16) was chosen for production of chimeric VLP in mg scale displaying the CSFV antigen as described below.

### Detection of target protein *N*-glycosylation

**Fig. 3:**
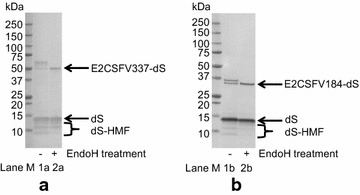
Protein deglycosylation assay of crude cell lysates derived from recombinant *H. polymorpha* strains D#53 (**a**) and D#73 (**b**) co-producing the dS and the fusion proteins E2CSFV337-dS or E2CSFV184-dS, respectively. Samples were analyzed by Western blot probed with anti-dS mAB 7C12 with (lanes 1a and 1b) or without (lanes 2a and 2b) previous EndoH treatment. M, molecular weight marker

Samples derived from each of the strains listed in Table 4 were subjected to the protein deglycosylation assay. Analysis of crude cell extract from strains D#53 and D#73 co-producing the dS and the fusion proteins E2CSFV337-dS or E2CSFV184-dS, respectively, are representatively shown in Fig. 3a, b. In both cases, one dominant dS-specific signal at approximately 14 kDa and therewith slightly below its theoretical MW (18.2 kDa) was accompanied with three weaker signals of higher mobility (dS-HMF). The comparisons of lanes 1a/b and 2a/b demonstrated that the dS-specific signals did not respond to EndoH treatment. This showed that the one potential *N*-glycosylation site present in the dS amino acid sequence was not occupied. Moreover, the occurrence of the dS-HMF could be influenced by treatment with different detergents indicating that they do not represent truncated species of the dS (data not shown).

**Table 4:** Summary of analytical results on target protein production and characterization

Designation of analyzed strains	Fusion protein co-produced with dS	*N*-Glycosylation of the fusion protein detected	Identity of foreign antigen confirmed	Chimeric VLP formation
D#113	E2BVDV344-dS	Yes	Yes	Yes
D#106	E2BVDV196-dS	Yes	Yes	Yes
D#117	dS-E2BVDV196	No	No	Yes
D#53	E2CSFV337-dS	Yes	Yes	Yes
D#73	E2CSFV184-dS	Yes	Yes	Yes
D#79	E2CSFV102-dS	No	Yes	Yes
M#22-8	FeLVp45-dS	Yes	NE	Yes
M#4-5	FeLVp45-dS and FeLVp15E-dS	NE	NE	Yes^a^
T#3-3	EDIIIWNV-dS	NE	Yes	Yes
Assay applied	Anti-dS Western blot	Protein deglycosylation assay	Western blot^b^	Ultracentrifugation

*NE* not examined

^a^Formation of chimeric VLP composed of dS, FeLVp45-dS and FeLVp15E-dS

^b^Applying primary antibodies specific for the respective foreign antigen as indicated in Table 1

In contrast, the fusion protein-specific bands were sensitive to treatment with EndoH. In the samples not treated with EndoH, the fusion proteins E2CSFV337-dS or E2CSFV184-dS appeared as clusters of distinct bands. Upon protein deglycosylation by incubation with EndoH (lanes 2a and 2b), the signals merged into one single band corresponding to the fusion protein-specific signal of lowest MW detected in lanes 1a or 1b, respectively. This indicated *N*-glycans cleaved off the fusion proteins and agrees with the presence of five or two potential *N*-glycosylation sites in the amino acid sequences. Glycosylation of the CSFV antigens demonstrated that they have been exposed to the lumen of the endoplasmic reticulum (ER) or Golgi system, the compartments of protein *N*-glycosylation. Analysis of the other designated strains producing the different fusion proteins is summarized in Table 4.

### Identity of the foreign antigens

**Fig. 4:**
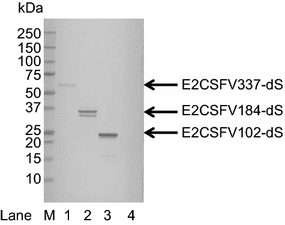
Western blot analysis of crude cell lysates probed with anti-CSFV E2 mAB. Samples originated from strains D#53 (lane 1), D#73 (lane 2) and D#79 (lane 3) co-producing the dS and the indicated fusion protein and A#299 (lane 4) producing the dS but no fusion protein. M, molecular weight marker

The identities of the foreign antigens were confirmed for all fusion proteins except the FelV-derived p15E-dS and p45-dS and the dS-E2BVDV196 by Western blot analysis applying monoclonal antibodies specific for the foreign antigens. In all cases the fusion proteins but not the unfused dS were detected. This is exemplarily shown in Fig. 4 for the three fusion proteins containing CSFV-derived antigens. For the fusion protein E2CSFV184-dS (lane 2), two distinct CSFV-specific signals were detected which corresponds to the anti-dS Western blot analysis (Fig. 3) and could be explained by the presence of *N*-glycans.

### VLP formation

Formation of chimeric VLP was analyzed by ultracentrifugation. For each of the strains listed in Table 4, the dS and the fusion protein accumulated in the boundary layer of the two sucrose solutions during sucrose cushion ultracentrifugation. Additionally, the target proteins isolated from this boundary layer were detected in the same fractions after subsequent CsCl density gradient ultracentrifugation. They were co-separated from *Hansenula* HCP due to lower density (1.1–1.2 g cm^−3^). Thus, chimeric VLP formation of the dS and every fusion protein co-expressed was indicated. Co-localization and co-separation from HCP during the ultracentrifugation steps was also observed for the three heterologous proteins dS, FeLVp45-dS and FeLVp15E-dS co-expressed in strain M#4-5. This indicated formation of a three-component chimeric VLP.

### Production of plain dS VLP using *H. polymorpha*

**Fig. 5:**
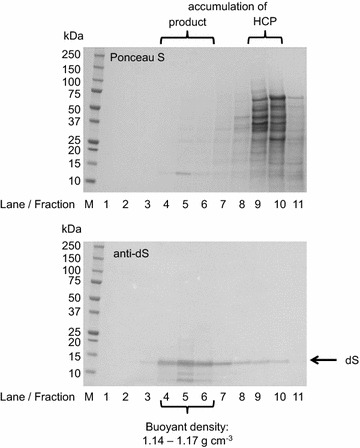
CsCl density gradient separation of HCP from plain dS VLP derived from recombinant *H. polymorpha* strain A#299 analyzed by Western blot. The ultracentrifugation tube was divided into 11 fractions with increasing density from fraction 1–11. Top: unspecific staining of proteins (Ponceau S). Bottom: anti-dS immunostaining using 7C12 mAB. M, molecular weight marker

**Fig. 6:**
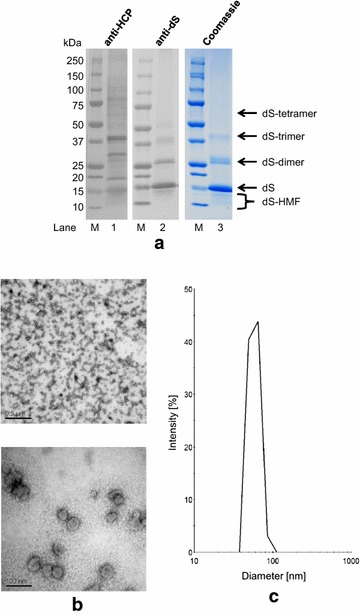
Analysis of plain dS VLP purified from recombinant *H. polymorpha* strain A#299. **a** Western blot probed with anti-HCP serum (10 µg protein loaded) or anti-dS mAB 7C12 (1 µg protein loaded), respectively and Coomassie stained PAA gel analysis (10 µg protein loaded); M, molecular weight marker; HMF, higher mobility forms. **b** TEM images at 50,000-fold (top) and 250,000-fold (bottom) magnification. **c** DLS data after regularization analysis

*Hansenula polymorpha* known as a potent host for recombinant HBsAg VLP production was shown to be well-suited for production of dS-based VLP. For demonstration and to set a benchmark for recombinant dS-based VLP production and purification, plain dS VLP were purified from strain A#299 in laboratory scale. Separation of the product from HCP contaminants in CsCl density gradient centrifugation is qualitatively shown in Fig. 5. This indicated the dS protein produced assembled into VLP structures characterized by a buoyant density of 1.14–1.17 g cm^−3^ which is consistent with a lipoprotein. Non-optimized fed batch fermentation yielded 35.2 ± 0.6 g L^−1^ dcw concentration during 70.5 h cultivation time. Using 70.4 ± 4 g dcw as biomass, 44.6 ± 2 mg VLP could be isolated. Analysis of the final preparation by SDS-PAGE followed by Coomassie staining or Western blotting, TEM imaging and DLS are shown in Fig. 6. Densitometric analysis of the Coomassie stained PAA gel (Fig. 6a, lane 3) indicated high purity (> 95%) which was confirmed by anti-HCP ELISA. This corresponds to a specific yield (Y_P/X_) of 0.63 ± 0.07 mg g^−1^ of dS VLP per biomass. In the purified sample, the dS subunits of the VLP appeared primarily as monomeric proteins with an apparent MW of 14–15 kDa which is slightly below its theoretical MW (18.2 kDa). However, dS oligomers with higher apparent MW as well as dS-HMF with lower apparent MW than the monomer were detected in Coomassie stained PAA gel and dS-specific Western blot.

The serum used in anti-HCP Western blot showed slight cross reactivity to the dS (Fig. 6a, lane 1). By loading 10 µg protein on the gel used for Western blotting, 14 individual HCP-specific signals could be detected by densitometry. DLS proved monomodal and monodisperse sample constitution (polydispersity index, PDI of 0.05) dominated by particles of 59 nm hydrodynamic diameter which was in good accordance with results from TEM imaging. Quantification of lipids yielded 0.79 ± 0.1 mg per mg protein which is equivalent to ~ 44% lipid content of the VLP.

### Production of chimeric VLP

The formation of chimeric VLP development could be demonstrated for all viral antigens summarized in Table 4 by analytical ultracentrifugation. Exemplarily, chimeric VLP were purified at several mg scale either from strain T#3-3 expressing EDIIIWNV-dS or strain D#79 expressing E2CSFV102-dS, respectively. The DSP for chimeric VLP purification was simplified compared to plain dS VLP production. Desorption of the product from the Aerosil matrix was allowed at RT and no ion exchange chromatography was performed prior to preparative CsCl density gradient centrifugation.

#### Chimeric VLP with EDIIIWNV-dS

**Fig. 7:**
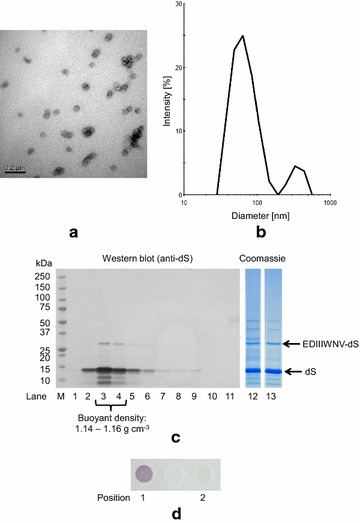
Characterization of chimeric VLP isolated from strain T#3-3 co-producing dS and EDIIIWNV-dS and desalted by SEC. **a** TEM image after negative staining (100,000-fold magnification); **b** DLS data after regularization analysis; **c** lanes 1–11: Western blot analysis of fractions harvested from analytical CsCl density gradient separation (density increases gradually from lanes 1–11) probed with anti-dS mAB 7C12; Coomassie stained PAA gels for analysis of final VLP preparations after desalting by dialysis (lane 12) or SEC (lane 13), 10 µg protein loaded; **d** dot blot analysis of the native sample desalted by SEC and probed with anti-WNV mAB, position 1: chimeric VLP displaying the WNV antigen, position 2: plain dS VLP as a negative control

For the chimeric VLP originating from strain T#3-3, dialysis and size exclusion chromatography (SEC) were compared for desalting after CsCl density gradient separation. SEC proved to serve as polishing step and increased VLP purity on protein level by about 9% to > 95% compared to desalting by dialysis based on analysis by densitometry of Coomassie stained gels (Fig. 7c, lanes 12 and 13). The specific product yield (Y_P/X_) was lowered by SEC by about 40% compared to desalting by dialysis from 0.7 to 0.4 mg chimeric VLP per g dcw. Based on the results obtained by densitometry, the chimeric VLP of both preparations consist of approximately 12% EDIIIWNV-dS and 88% wild-type dS. The chimeric VLP originating from SEC were characterized in more detail by TEM, DLS, CsCl density gradient separation and in dot blot assay as shown in Fig. 7. Analysis by TEM confirmed the formation of particles of predominantly 40–50 nm according to manual evaluation. Size distribution analysis by DLS detected a dominating particle population characterized by a hydrodynamic diameter of 67 nm (PDI 0.13) which corresponds well with the TEM imaging analysis. As expected for chimeric VLP, the dS and the fusion protein EDIIIWNV-dS accumulated in the same fractions during CsCl density gradient centrifugation with plausible buoyant densities for lipoproteins of 1.14–1.16 g cm^−3^.

Additionally, the display of the WNV antigen on the VLPs’ surface was shown by dot blot analysis under native conditions.

#### Chimeric VLP with E2CSFV102-dS

**Fig. 8:**
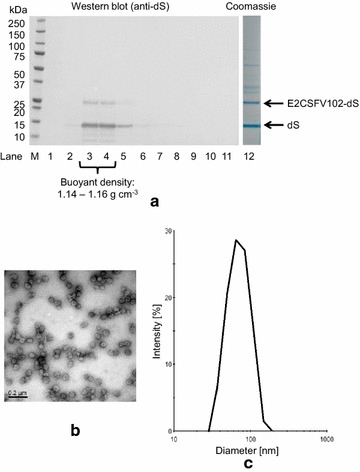
Evaluation of chimeric VLP formation from material originated from strain D#79 co-producing dS and E2CSFV102-dS. **a** Lanes 1–11: Western blot analysis of fractions harvested from CsCl density gradient separation (density increases gradually from lanes 1–11), probed with anti-dS mAB 7C12; Lane 12: Coomassie stained PAA gel of pooled and desalted fractions 3 and 4. **b** TEM image after negative staining (100,000-fold magnification); **c** DLS data after regularization analysis

**Fig. 9:**
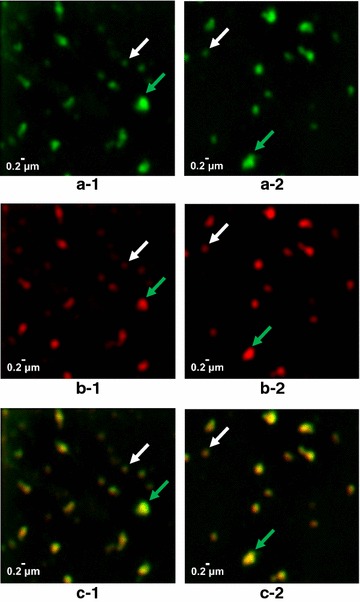
Chimeric VLP isolated from strain D#79 composed of dS and E2CSFV102-dS were analyzed under native conditions by N-SIM. Two series of images obtained from the same sample are presented showing fluorescence immunolabeling of dS in green (**a-1**, **a-2**), CSFV E2 antigen in red (**b-1**, **b-2**) and co-localization of the two labels in superimposed images in yellow (**c-1**, **c-2**). In each series of images two spots were consistently marked by arrows: signals of the size expected for individual VLP (white); largest signals in the respective frame (green)

Processing cell paste from strain D#79 yielded 0.5 g chimeric VLP per g dcw composed of approximately 33% E2CSFV102-dS and 67% wild-type dS according to analysis by densitometry of a Coomassie stained PAA gel (Fig. 8a, lane 12). Purity of the chimeric VLP preparation was ~ 75% based on the same analysis. The behavior of the VLP displaying the CSFV antigen in CsCl density gradient separation (Fig. 8a) was highly comparable to that of plain dS VLP (Fig. 5) and chimeric VLP displaying the WNV antigen (Fig. 7a): the two target proteins accumulated in the same fractions with buoyant densities of 1.14–1.16 g cm^−3^. Integrity of the VLP was demonstrated by TEM imaging detecting spherical particles of predominantly 50–70 nm. This was in good correlation with the size distribution analysis by DLS which verified monodisperse monomodal sample constitution characterized by a hydrodynamic diameter of 73 nm and a PDI of 0.11. Additionally, super-resolution imaging by N-SIM using specific antibodies demonstrated the accessibility of dS (Fig. 9a) and CSFV E2 (Fig. 9b) epitopes under native conditions. The signals from labeling each of dS and CSFV E2 co-localized in the same nano-scale structures (Fig. 9c) suggesting the display CSFV antigen on the particles’ surface. The SIM system was calibrated with 100 nm fluorescent beads (TetraSpeck™ microspheres, Thermo Fisher Scientific, Waltham, USA) which showed an apparent diameter of about 150 nm in the raw images (data not shown). The detected structures in Fig. 9 were either round shaped and of about 250 nm in diameter (representatively marked by white arrows) or larger (400–800 nm, marked by green arrows) and irregular shaped. Complexes of primary and secondary antibody were reported to stretch out for up to 30 nm [65] which may result in a larger apparent diameter of antibody decorated individual VLP.

### Stability assessment of chimeric VLP

**Fig. 10:**
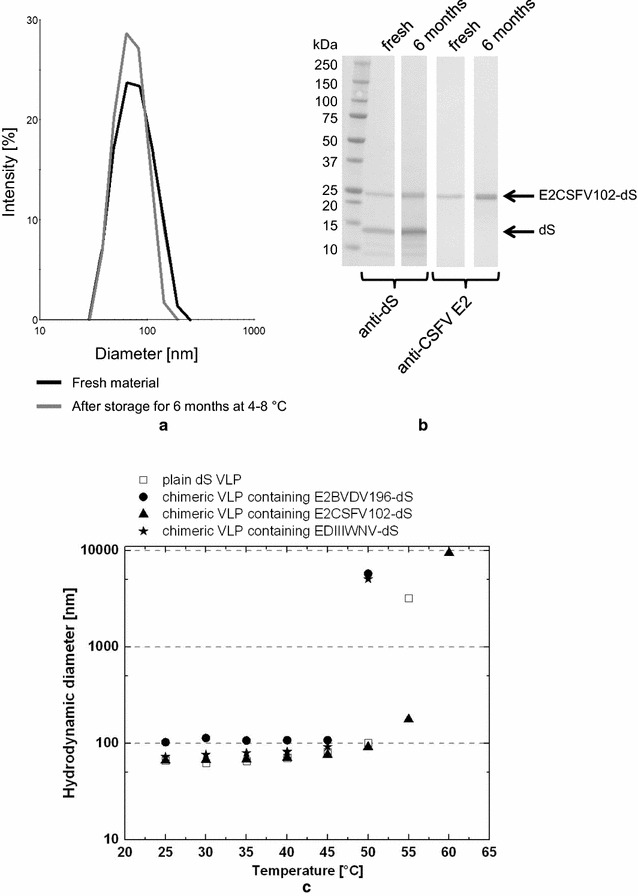
Stability assessment of chimeric VLP. **a**, **b** DLS and Western blot analysis of real time stability experiment of purified VLP composed of dS and E2CSFV102-dS, isolated from strain D#79 and formulated at mg mL^−1^ protein concentration in desalting buffer. Data of fresh sample analysis are compared to data collected after 6 months of storage at 4–8 °C by independent but volume-normalized Western blot analysis. **c** DLS analysis of plain dS VLP and different chimeric VLP analyzed during step-wise increasing temperature allowing 5 min equilibration time in between the measurements

Shelf life and stability are generally critical for biotechnological products. Especially in the case of chimeric VLP incorporating large foreign antigens, VLP integrity over time or at elevated temperatures appears questionable. Both aspects were therefore investigated separately in our study (Fig. 10). Chimeric VLP isolated from strain D#79 and composed of dS and E2CSFV102-dS were analyzed by DLS and Western blot (Fig. 10a, b) immediately after preparation (“fresh”) and after 6 months of storage at 4–8 °C. Both methods manifested only minor changes of the samples’ constitution: A slight increase of hydrodynamic diameter (from 73 to 82 nm) as well as PDI (from 0.11 to 0.14) could be verified by DLS. Additionally, the fusion protein-specific signal in anti-dS and anti-CSFV E2 Western blot appeared more diffuse or as a pair of bands running closely to each other in the sample stored for 6 months compared to the freshly analyzed sample. However, loss of particulate character or substantial degradation of the product proteins was not observed.

Thermal stability was tested by DLS for four different VLP preparations (Fig. 10c): plain dS VLP and chimeric VLP containing E2BVDV196-dS, E2CSFV102-dS or EDIIIWNV-dS. The determined hydrodynamic diameter of each of the different VLP changed only marginally during step-wise increase of the chamber temperature from 25 to 45 °C. For plain dS VLP and chimeric VLP containing E2CSFV102-dS or EDIIIWNV-dS, the diameter appears to slightly increase with increased temperature. This is most likely due to enhanced VLP collisions and therewith apparently reduced speed of Brownian motion. However, pronounced increase in hydrodynamic diameter indicating onset of VLP deformation or aggregation could be observed upon temperature increase from 45 to 50 °C for all VLP preparations. Complete aggregation occurred at 50 °C for chimeric VLP containing E2BVDV196-dS or EDIIIWNV-dS, at 55 °C for plain dS VLP or at 60 °C for chimeric VLP having the E2CSFV102-dS fusion protein incorporated, respectively.

## Discussion

In this work, VLP formed by the dS were shown to be an effective platform for rational development of chimeric VLP displaying a variety of large foreign antigens. For the establishment of a robust platform, the methylotrophic yeast *H.* *polymorpha* proved to perform as a reliable microbial cell factory; none of the constructed fusion proteins failed to be co-expressed with the dS. During recombinant production, the fusion proteins were shown to be exposed to the lumen of the yeasts’ ER or Golgi system. They accumulated intracellularly and carried *N*-glycans if they had potential *N*-glycosylation sites within their amino acid sequence. Based on this, it can be assumed that the mechanism of dS-based VLP formation in recombinant *H.* *polymorpha* is highly comparable to the morphogenesis of HBsAg VLP in methylotrophic yeast *Pichia pastoris* [66]. The product proteins presumably accumulate in the yeasts’ subcellular membrane structures and congregate during DSP to plain dS VLP or chimeric VLP, respectively.

Chimeric VLP formation required co-production of the dS and a fusion protein in a single recombinant host. Therefore, a toolbox of strain generation strategies independent of antibiotic resistance genes was established. Isolation of heterologous *H. polymorpha* strains stably co-producing the heterologous proteins was allowed within only a single sequence of yeast transformation and subsequent strain selection allowing fast and simple generation of recombinant strains. Production levels of the respective fusion protein and the dS were observed to differ among the isolated yeast strains. Especially in the case of the fusion protein E2CSFV102-dS, the efficiency of chimeric VLP solubilization during cell disruption was found to be dependent on the relative expression levels of the fusion protein and the dS. The reason for reduced efficiency of target protein solubilization in case of higher relative amounts of the fusion protein remains ambiguous. It can be argued that the solubilization of dS and E2CSFV102-dS strongly depended on chimeric VLP formation since both proteins are membrane spanning and thus rather unlikely to be solubilized as monomers during cell disruption. It is believed that an excess of dS over the fusion protein is essential for chimeric dS-based VLP formation [13, 14] although detailed studies on this have not yet been published. Probably, incorporation of foreign antigens into the dS VLP scaffold is limited by steric issues which may arise if the density of foreign antigens within a VLP-forming structure exceeds a certain threshold. Interestingly, in these cases formation of dS VLP without or with low relative amounts of E2CSFV102-dS was not observed. This indicated that the two target proteins accumulated intracellularly in close proximity to each other and interacted with one another prior to cell lysis. However, the observed variety among the isolated and characterized production strains (Fig. 2), allowed us to pick the strain best suited for the integration into the DSP [31].

No protein purification tags were used during DSP which is highly desired for most applications especially for vaccines or pharmaceutical products [67]. Also, cost-intensive steps like immunoaffinity chromatography were not required here. Nevertheless, elimination of the costly CsCl density gradient purification step appears desirable to further improve cost efficiency.

Processing of cell paste from strain A#299 yielded plain dS VLP of similar quality (> 95% purity) and yield per biomass (0.63 ± 0.07 mg g^−1^) compared to literature on HBsAg VLP purification (~ 0.6 mg g^−1^, [68]). However, final recovery per culture volume was lower (22.3 ± 2 vs. ~ 50 mg L^−1^) due to non-optimized fermentation procedure applied in this study in contrast to carefully optimized fermentation protocol for HBsAg VLP production [69]. Since *H.* *polymorpha* is well known to be industrially applicable and to grow beyond 100 g dcw L^−1^ culture volume [70], improvements regarding the volume-normalized product yield can be expected after fermentation optimization. Additionally, the use of a synthetic growth medium during fermentation is highly desirable regarding regulatory approval for production of bio-pharmaceuticals [71]. The lipid content (~ 44%), the dimensions (59 nm hydrodynamic diameter) and the buoyant density (1.14–1.17 g cm^−3^) of *Hansenula*-derived dS VLP showed high similarity to what is described for natural occurring DHBV VLP (30–40%; 35–60 nm; 1.14–1.6 g cm^−3^) [20, 72].

Chimeric VLP presenting different foreign antigens could be purified by applying basically the same DSP that was used for purification of dS VLP before with similar product yields and protein purity. The purified chimeric VLP contained 33% E2CSFV102-dS or 12% EDIIIWNV-dS respectively, which is reasonable in the context of chimeric VLP vaccines [73]. We can only speculate about the number of VLP-forming protein subunits per individual VLP. The spherical ~ 22 nm HBV VLP contain approximately 100 HBsAg molecules [74]. Thus, dS-based VLP presumably contain well over 100 protein subunits due to their larger dimensions.

The foreign antigens of both chimeric VLP preparations containing either EDIIIWNV-dS or E2CSFV102-dS were shown to be accessible for immunolabeling under native conditions (Figs. 7d, 9). These assays suggest surface exposure of the foreign antigens on VLP. In addition, analysis by N-SIM demonstrated co-localization of the fusion protein and the dS in the same nano-scale particles. While the resolution may not be sufficient to localize both proteins in individual VLP, the authors conclude that co-localization in structures representing clusters of few VLP would support the presence of both proteins in individual VLP due to the physicochemical homogeneity of the analyzed sample.

Thermal stability of the recombinant VLP preparations was demonstrated (Fig. 10c) and could be explained by high similarity in their physicochemical properties compared to the native DHBV VLP. Since VLP are complex structures, multiple factors like mode of VLP purification, type and content of fusion protein and lipid content probably affect thermal stability which precludes simple explanation of the slight differences detected by this analysis (Fig. 10c). Preliminary 6 months real time stability data of chimeric VLP in simple PBS-like buffer support the use of this platform for vaccine development purposes. However, the potential application of the developed chimeric VLP as veterinary vaccine candidates cannot be shown without immunization and animal challenge studies. This represents the key task for the near future to extend the antigen presentation platform into a vaccine development platform.

## Conclusions

This study describes the establishment of a robust and versatile VLP platform for presentation of large antigens. Based on the methylotrophic yeast *H.* *polymorpha*, it allows rational design, cost-effective production and purification of chimeric VLP. A variety of antigens originating from different animal-infecting viruses and described as highly immunogenic was successfully incorporated into a stable VLP scaffold formed by the dS. The obtained product yields make this technology a seriously competitive VLP development platform that should be considered for veterinary DIVA vaccine development in the future.
